# Prospects and limits of the flow cytometric seed screen – insights from *Potentilla sensu lato* (Potentilleae, Rosaceae)

**DOI:** 10.1111/nph.12149

**Published:** 2013-02-21

**Authors:** Christoph Dobeš, Andrea Lückl, Karl Hülber, Juraj Paule

**Affiliations:** 1 Department of Pharmacognosy, Pharmacobotany, University of ViennaAlthanstrasse 14, A–1090, Vienna, Austria; 2 Department of Conservation Biology, Vegetation and Landscape Ecology, University of ViennaRennweg 14, A–1030, Vienna, Austria; 3 Vienna Institute for Nature Conservation & AnalysesGiessergasse 6/7, A-1090, Vienna, Austria; 4 Department of Botany and Molecular Evolution, Senckenberg Research Institute & Biodiversity and Climate Research Centre (BiK-F)Senckenberganlage 25, D–60325, Frankfurt am Main, Germany

**Keywords:** apomixis, cytology, embryo, endosperm, flow cytometry, gametes, *Potentilla*, reproduction

## Abstract

- The flow cytometric seed screen allows for identification of reproductive modes of seed formation and inference of the ploidy of contributing gametes. However, the lack of a mathematical formalization to infer male/female genomic contributions, and the prerequisite of a binucleate female contribution to the endosperm limits its applicability.
- We evaluated this assumption combining a DNA-based progeny survey with a comparison of the cytology of reproductive pathways co-occurring within single individuals representing 14 Potentilleae species from six phylogenetic lineages.
- A numerical framework valid for sexual and pseudogamous taxa was developed, enabling quantification of female and male genomes contributing to embryo and endosperm independent of gametophyte origins, numbers of sperm involved and ploidy of parents. The inference strongly depended on accurate peak index estimation. The endosperm of Potentilleae species received a binucleate female contribution in five evolutionary lineages whereas endosperm formation remained uncertain in the Tormentillae. A modified flow cytometric seed screen protocol was developed to cope with low endosperm contents.
- Evolutionary conservation of a binucleate female contribution to the endosperm suggested wide applicability of flow cytometric seed screen – at least in the Potentilleae. However, alternative progeny surveys and precise embryo/endosperm ploidy estimates are required for a comprehensive understanding of the cytology of seed formation.

## Introduction

Since its introduction (Matzk *et al*., 2000) the flow cytometric seed screen (FCSS) has become an increasingly important tool for the identification of reproductive pathways of seed formation in angiosperms. The method provides information on the origin of the embryo sac (i.e. female gametophyte), the embryo and the endosperm by estimating the DNA content (or DNA-ploidy) of the endosperm and embryo. Thus, FCSS allows differentiation between the meiotic and the apomeiotic formation of the embryo sac, the parthenogenetic and zygotic origin of the embryo and the autonomous vs pseudogamous development of the endosperm. Based on the combination of these processes, as well as variation in the male genomic contribution, seven principal reproductive pathways were identified with regard to the regular eight-nucleate embryo sac of vascular plants (Matzk *et al*., 2000). Each pathway is characterized by a specific combination of embryo and endosperm ploidies. The correspondence, however, depends on the contribution of two polar nuclei to the endosperm.

In the Panicoideae (i.e. some *Panicum* and *Pennisetum* species) of the Poaceae only one polar nucleus contributes to the endosperm in apomicts and contrasts with a binucleate contribution in the sexual relatives (Rutishauser, 1969; Crane, 2001). In some Rosaceae (e.g. *Amelanchier* Medik., *Cotoneaster* Medik., Maloideae) an association of reproductive mode with the number of polar nuclei involved in endosperm formation was suggested based on the fusion of the polar nuclei in sexual species but not in their apomictic relatives (Hjelmqvist, 1962; Campbell *et al*., 1985, 1987; summarized by Talent & Dickinson, 2007). Similarly, Czapik (1983, 1985a) hypothesized that an early fusion of polar nuclei is correlated with sexual reproduction, while the retardation of the fusion is connected to apomixis in the Rosoideae genera *Rubus* L. and *Waldsteinia* Willd., leaving the option of a uninucleate female contribution to the endosperm in apomicts. For taxa receiving a uninucleate and binucleate female contribution to the endosperm of apomictically and sexually derived seeds, respectively, FCSS fails to differentiate between these reproductive modes as, in both cases, diploid embryos and triploid endosperms are formed (Matzk *et al*., 2000). A major methodological caveat in the determination of numbers of contributing polar nuclei by applying cyto-embryological techniques is, however, the difficulty in observing the moments of fusion of polar and sperm nuclei and to simultaneously establish the (meiotic vs apomeiotic) origin of embryo sacs (Czapik, 1985b).

The FCSS has also been used to assess the ploidy of female and male gametes contributing to the embryo and endosperm (Barcaccia *et al*., 2006; Talent & Dickinson, 2007; Krahulcová *et al*., 2009). Thereby, gamete ploidy was inferred by comparing empirical embryo/endosperm ploidies with those expected for simple cytological models of seed formation (Hörandl *et al*., 2008). For example, haploid sperm and haploid egg cells are expected for seeds with diploid embryos and triploid endosperm. However, there is no mathematical formalization of the inference of gamete ploidies from embryo and endosperm ploidies. Thus, from these empirical studies, it remains unknown whether methodological inaccuracies of embryo and endosperm ploidy estimation interferes with the inference or whether aneuploid changes in chromosome number occurring in one or, in particular, simultaneously in both gametes can be quantified.

The applicability of FCSS is limited by several factors, including (among others): the occurrence of secondary chemical compounds interfering with DNA staining (Jedrzejczyk & Sliwinska, 2010); the efficiency of nuclei extraction; the occurrence of nuclei in G2 phase and endopolyploidy masking the endosperm peaks (Krahulcová & Rotreklová, 2010); and insufficient amounts of endosperm tissue. Despite these problems, reproductive modes were inferred using FCSS in a number of apomictic angiosperms, in particular the Asteraceae, Brassicaceae, Poaceae, *Ranunculus* and *Hypericum* (Matzk, 2007; Hörandl *et al*., 2008; Krahulcová & Rotreklová, 2010). Within the Rosaceae, FCSS has been applied to the genus *Crataegus* (Talent & Dickinson, 2007) and recently to a few seeds of two *Potentilla* species (Hörandl *et al*., 2011). However, FCSS has so far failed in most *Potentilla* species (Dobeš unpubl.; Matzk *et al*., 2000) most likely because of the low (Corner, 1976) or reduced (Martin, 1946) amount of endosperm tissue or the absence of endosperm (Kalkman, 2004) in mature seeds. Thus, the general applicability of FCSS in *Potentilla* and related genera remains to be verified.

The genus *Potentilla* constitutes one of the most diverse plant genera in the Northern Hemisphere comprising *c*. 300 (Wolf, 1908) to 500 species (Airy Shaw, 1973). Molecular phylogenetic reconstructions of the tribe Potentilleae, revealed a diversification of *Potentilla sensu lato* into several distinct evolutionary lineages now considered as segregate genera (i.e. *Argentina*, *Comarum*, *Dasiphora*, *Drymocallis*, *Sibbaldia*; Eriksson *et al*., 1998). The radiation of *Potentilla sensu stricto* was inferred to have started *c*. 17–25 million yr ago and gave rise to a major northern hemispheric lineage (the ‘core Potentillas’) representing the vast majority of the species described, as well as other geographically restricted clades of limited taxonomic diversity (Dobeš & Paule, 2010). The phylogeny provides a taxonomic and evolutionary framework in which the distribution and variability of reproductive modes appear to be of particular importance. Apomixis seems to be mainly present in the core Potentillas, but data on reproductive modes and the cytology of seed formation are largely missing for the most closely related genera.

Reproductive modes in the genus *Potentilla* L. have been mainly studied using traditional techniques involving microdissections of ovaries and controlled crosses. Based on embryological studies, at least one single element of apomixis (i.e. apomeiosis or parthenogenetic embryo development) was observed in *c*. 16 *Potentilla* species (Gentscheff, 1938; Gustafsson, 1947; Löve, 1954; Asker, 1970a; Nyléhn *et al*., 2003). Regular sexual production has been reported for five species (Rutishauser, 1945; Håkansson, 1946; Czapik, 1961, 1962). Embryo sacs are usually eight-nucleate, as is commonly the case in angiosperms (Rutishauser, 1967), but a five-nucleate embryo sac has also been recorded (Eriksen & Fredrikson, 2000). In contrast to female gametogenesis, pollen is almost exclusively reduced because of regular meiosis (Müntzing, 1928; Rutishauser, 1943; Asker, 1970b, 1985). In all documented cases functional pollen is needed to initiate seed formation in both sexual species and apomicts, suggesting the general occurrence of pseudogamy in *Potentilla* (Asker, 1970b). However, evidence for the role of pollen in seed production comes mainly from controlled crosses (e.g. Müntzing, 1928; Asker, 1970a,b).

Embryological observations on the origin and cytology of the endosperm are, by contrast, rare (Czapik, 1996) and limited to a few *Potentilla* species from a single phylogenetic lineage. Pentaploid (Gentscheff & Gustafsson, 1940; Håkansson, 1946; Smith, 1963b) and triploid (Czapik, 1962) endosperms were recorded in few cases and suggested a 4 : 1 and 2 : 1 female: male genomic contribution in apomicts and sexual species, respectively. These ratios indicated the involvement of two polar nuclei and a single reduced sperm in the formation of the endosperm. In accord with this interpretation, degeneration of one sperm was observed in an apomictic individual (Håkansson, 1946). The presence and fusion of two polar nuclei was observed in both apomictic (Gentscheff & Gustafsson, 1940; Håkansson, 1946; Smith, 1963a) and sexual (Czapik, 1961) *Potentilla* species. Interestingly, occasional involvement of more than two polar nuclei in the formation of the central cell (Håkansson, 1946; Smith, 1963a) and fertilization of a single polar nucleus by one sperm (Czapik, 1961) have also been observed.

In the present paper we present a mathematical formalization suitable for all pseudogamous taxa to calculate the male and female genomic contribution to the embryo and endosperm irrespective of the mode of male and female gametophyte formation (i.e. meiotic vs apomeiotic), the number of involved sperm and the ploidy of parents. The new formulae are applicable as long as both polar nuclei contribute to the endosperm (i.e. binucleate female contribution). To provide the basic prerequisite, we introduce a comprehensive theoretical framework of hypothetical cytological pathways of seed formation under the experimental conditions of equal ploidy of parents. Twenty pathways can be defined as a unique combination of five variable cytological elements of seed formation, but represent only 16 unique combinations of embryo and endosperm ploidy. Thus, the cytology of most but not all pathways can be unambiguously determined by FCSS. To discriminate pathways with identical embryo/endosperm ploidies but differing in the number of polar nuclei involved, we propose an amplified fragment length polymorphism (AFLP)-based progeny survey and a comparison of cytological elements of nondistinguishable pathways with that of pathways of known cytology co-occurring in individuals based on the principle of parsimony.

Specifically, we: (1) verify the applicability of FCSS in the tribe Potentilleae, including the establishment of a suitable laboratory protocol; (2) introduce a theoretical model that relates the hypothetically anticipated embryo/endosperm ploidies to variation in male and female genomic contributions – including variable numbers of polar nuclei; (3) infer reproductive modes in selected taxa representing the major phylogenetic lineages of the Potentilleae; and (4) explain derivation of the formulae, explore their mathematical properties and discuss associated consequences for the cytological interpretation of the FCSS results.

## Materials and Methods

### Plant material

Seven genera and 14 species from the tribe Potentilleae were studied: *Argentina* (one species), *Comarum* (one species), *Dasiphora* (one species), *Drymocallis* (one species), *Horkeliella* (one species), *Potentilla* (seven species), and *Sibbaldia* sensu Soják (2008) (two species). The taxa represent six out of the nine major phylogenetic lineages of the Potentilleae distinguished by Dobeš & Paule (2010) (Table 1). One accession per species was analysed. Vouchers are deposited in the herbaria HEID, W, and WUP. Mature fruitlets were collected from single plants and analysed within 3 months. Plants were bagged before anthesis to exclude cross-fertilization from related species or cytotypes.

**Table 1:** Geographic origin and phylogenetic position of seven genera and 14 species from the Potentilleae studied

Taxon	Clade	Ploidy	Individual	Origin and collection history, herbarium specimen
*Argentina anserina* (L.) Rydb.	B	4*x*^a^	Ptl8415	Austria, Vienna, Neuwaldegg, 1.385 km NNW of the Heuberg, 16.270556° E/48,238056° N, 290 m asl, leg. 21.08.2011 Christoph Dobeš, W 2012-03009
*Comarum palustre* L.	A	6*x*^a^	Ptl8410	Austria, Salzburg, Schladminger Tauern, Prebersee, 13.85667° E/47.18333° N, 1514 m asl, leg. 05.08.2011, Johannes Saukel, W 2012-02520
*Dasiphora fruticosa* (L.) Rydb.	A	2*x*^b^	Ptl8411	Canada, Ontario, Bruce County, Lindsay Tp., Pleasant Harbour, 82.46667° E/45,03333° N, 183 m asl, leg. The Arboretum University of Guelph, W 2012-02576
*Drymocallis arguta* Pursh	A	2*x*^a^	Ptl2650	Garden accession, leg. Botanical Garden Bayreuth 708, HEID 806320
*Horkeliella purpurascens* (S. Watson) Rydb.	E	8*x*^a^	Ptl8418	USA, California, Kern County, Squirrel Meadow on Breckenridge Mountain *c*. 13 airmiles SSW of Lake Isabella, 118.578° W/35.475° N, leg. Barbara Ertter 20764, W 2012-02903
*Potentilla argentea* L.	C	6*x*^b^^,^^c^	Ptl3126	Sweden, Gotland, NE Klintehamn, 18.166083° E/57.3966° N, leg. 05.06.2006 Thomas Gregor, HEID 804627 to 804629
*Potentilla calabra* Ten.	C	2*x*^b^	Ptl4731	Italy, Calabria, between Germano and the junction of Longobucco/Bocchigliero/Lago di Cecita, 16.6239° E/39.371416° N, 1570 m asl, leg. 08.06.2007 Christoph Dobeš, W 2012-02429
*Potentilla incana* Gaertn. Mey. & Scherb.	C	4*x*^b^	Ptl8249	Italy, Abruzzo, Monti della Laga, summit of the Monte Gemella, 13.60044° E/42.763472° N, 1790-1810 m asl, leg. 27.06.2011 Christoph Dobeš, W 2012-02872
*Potentilla indica* L.	F	12*x*^a^	Ptl8202	Austria, Vienna, weed in the medicinal garden of the Department of Pharmacognosy, University of Vienna, 16.35972° E/48.23278° N, 200 m asl, leg. 27.05.2011 Christoph Dobeš, W 2012-02872
*Potentilla micrantha* Ramond ex DC.	G	2*x*^c^	Ptl8224	Italy, Calabria, Sila Piccola, ESE of the Monte Gariglione, 16.67867° E/39.1295° N, 1640 m asl, leg. 04.06.2011 Christoph Dobeš, W 2012-02417, -02873
*Potentilla norvegica* L.	C	10*x*^a^	Ptl2714	Germany, Bremen, harbour, 8.7725° E/53.09444° N, leg. Botanical Garden Göttingen, HEID 806384 to 806390
*Potentilla thuringiaca* Bernh.	C	9*x*^b^	Ptl4606	Switzerland, Grisons, Engadin, between Ftan-Pitschen and Scoul, 10.260833° E/46.7942167° N, 1620 m asl, leg. 13.05.2007 Christoph Dobeš, W 2012-02162
*Sibbaldia procumbens* L.	A	2*x*^c^	Ptl8407	Austria, Salzburg, Schladminger Tauern, Kleines Gurpitscheck, 13.62222° E/7.20778° N, 2100 m asl, leg. 03.08.2011 Johannes Saukel, W 2012-02573
*Sibbaldia tridentata* (Aiton) Paule & Soják (= *Sibbaldiopsis tridentata* [Aiton] Rydb.)	A	–d	Ptl8413	Canada, leg. Botanical Garden Montreal, W 2012-02578

‘Clade’ refers to phylogenetic lineages according to Dobeš & Paule (2010). ‘Ploidy’ specifies the generative ploidy of the studied accession. Coordinates are given in WGS-84 standard.

a New chromosome count.

b Flow cytometric inference using reference individuals of known ploidy.

c Literature record.

d Polyploid according to the literature.

### Flow cytometric seed screen

The testa and the fruit wall of fruitlets were removed before flow cytometric analyses. The FCSS was performed separately for each of 477 seeds with fully developed, nondegraded fleshy embryos. Twenty to 47 seeds were analysed per accession. *Pisum sativum* cv Kleine Rheinländerin and a tetraploid strain of *Potentilla puberula* Krašan (Ptl8148) co-chopped with the sample served as internal standards. We used DAPI (4′-6-diamidino-2-phenylindole) and alternatively propidium iodide (PI) as DNA-selective stains. Samples were prepared using a two-step procedure and Otto I + II buffers (Otto, 1990) according to Matzk *et al*. (2000) with the following modifications applied: the Otto II buffer contained 40 ng DAPI ml^−1^ only or 50 μg PI ml^−1^. Suitable seeds were chopped in 200 μl extraction buffer. Three hundred microlitres of extraction buffer was subsequently added to the cut sample and the Petri dish containing the homogenate was placed on ice. After a delay of *c*. 30 min the Petri dish was carefully placed in an ultrasonic bath and ultrasonicated for 1 min. The sample was filtered through a 20 μm nylon mesh filter (Partec CellTrics, Partec Münster, Germany). The PI samples were RNA-digested for 30 min at 37°C by applying 50 μl RNase A solution (3 mg ml^−1^) per sample added to 1 ml of Otto II buffer. The DAPI-stained samples and PI-stained samples were measured after 5 min and 45 min, respectively, on CyFlow Ploidy Analyser instruments (Partec, Münster, Germany) equipped with a 365 nm LED and a 532 nm green laser. Signals other than nuclei were rejected using the side scatter. The embryo to standard fluorescence ratio and the peak index (i.e. the endosperm to embryo fluorescence ratio) were calculated from the means of the fluorescence histograms. As a measure of the relative number of endosperm nuclei in a sample, we defined the count ratio as the percentage of counts for the endosperm peak relative to that for the embryo peak.

### Ploidy level estimation

The ploidy level of mother plants for species with uniform chromosome number was taken from the literature (Moore, 1970; Goldblatt, 1981). The chromosome number of the other species was determined in root tips of mother plants following Dobeš (1999). Alternatively, DNA ploidy levels (Suda *et al*., 2006) were established by comparison of the sample to standard fluorescence ratios of mothers with the ratios of individuals karyotyped elsewhere (Dickson *et al*., 1992; Paule *et al*., 2011, 2012; Dobeš *et al*., 2012). For convenience we henceforth refer to the measured DNA ploidy as ploidy. Sample to standard fluorescence ratios of mothers were averaged over three repeated measurements of leaf petioles applying the protocol used by Paule *et al*. (2011) and compared with the equivalent ratio of embryos and seedlings used in the progeny survey (see ‘Progeny survey’ section) to determine the ploidy of the latter. The male and female genomic contributions to the embryo and the endosperm (i.e. number of genomes transferred by the egg cell, polar nuclei and sperm), respectively, were calculated from the peak index and embryo ploidy using the formulae developed within this study and detailed in the Supporting Information, Notes S1.

### Inference of cytological pathways of seed formation

Reproductive pathways were inferred based on a theoretical concept assuming variation in the following five cytological elements of seed formation: meiotic vs apomeiotic origin of the female gametophyte (i.e. embryo sac) and meiotic vs apomeiotic origin of the male gametophyte; parthenogenetic vs zygotic development of the embryo; contribution of one or two polar nuclei; and contribution of one or two sperm to the endosperm (Table 2). Equal ploidy of mother plants and pollen donors is assumed, which was ensured by the bagging of flowers. The combination of the five cytological elements results in 24 hypothetical pathways of seed formation. In four cases two pathways differ only in the way two holoploid male genomes are contributed to the endosperm, that is, the 2*n* male contribution is alternatively achieved by two reduced (*n* + *n*) or one unreduced (2*n*) sperm (we use *n*, the haplophasic chromosome number, to indicate the number of holoploid genomes and *x*, the chromosome number of the monoploid genome, when referring to the generative ploidy; see Greilhuber, 2005). Each of these pairs of pathways were merged and thereupon considered as a single pathway (*C*, *F*, *G*, and *K* in Table 2), reducing the total number of pathways to 20. These pathways correspond to 11 peak indices. Pathways referring to identical peak indices can be further distinguished based on difference in embryo ploidy except for four pairs. Pathways of two of these pairs (*E*/*F* and *G*/*H*) differ in the cytology of endosperm formation (i.e. the origin of the male gametophyte and number of polar nuclei and sperm involved; Table 2) but describe biologically similar reproductive modes (i.e. haploid parthenogenesis and apomixis). By contrast, the pairs *M*/*O* and *R*/*S* each comprise pathways of biologically unlikely relevance. Pathways *M* and *O* refer to regular sexual vs apomictic reproduction and pathways *R* and *S* refer to sexual reproduction involving an unreduced sperm vs an unreduced egg cell.

**Table 2:** Hypothetical pathways of seed formation in facultative pseudogamous apomicts assuming variation in the following cytological elements: meiotic vs apomeiotic origin of the female gametophyte (i.e. embryo sac); meiotic vs apomeiotic origin of the male gametophyte; parthenogenetic vs zygotic development of the embryo; contribution of one or two polar nuclei; and one or two sperm to the endosperm

Peak index	pw	Gametophyte origin		Embryo development	Endosperm origin		Embryo/endosperm ploidy (both for ♀ + ♂)	Summary of events
	
		♀	♂		*N* polar nuclei	*N* sperm		
6	*A*	M	Apo	Parthenogenetic	2	2	1*n* + 0*n*/2*n* + 4*n*	Haploid parthenogenesis, 4*n* ♂-contribution
5	*B*	M	Apo	Parthenogenetic	1	2	1*n* + 0*n*/1*n* + 4*n*	
**4**	*C*	M	M/Apo	Parthenogenetic	2	2/1^a^	1*n* + 0*n*/2*n* + 2*n*	Haploid parthenogenesis, 2*n* ♂-contribution
	***D***	**Apo**	**Apo**	**Parthenogenetic**	**2**	**2**	**2*****n*** **+ 0*****n***/**4*****n*** **+ 4*****n***	**Apomictic, 4*****n*** **♂-contribution**
**3**	***E***	**M**	**M**	**Parthenogenetic**	**2**	**1**	**1*****n*** **+ 0*****n***/**2*****n*** **+ 1*****n***	**Haploid parthenogenesis, 1*****n*** **♂-contribution**
	*F*	M	M/Apo	Parthenogenetic	1	2/1^a^	1*n* + 0*n*/1*n* + 2*n*	Haploid parthenogenesis, 2*n* ♂-contribution, 1 polar nucleus fertilised
	***G***	**Apo**	**M/Apo**	**Parthenogenetic**	**2**	**2/1**^a^	**2*****n*** **+ 0*****n***/**4*****n*** **+ 2*****n***	**Apomictic, 2*****n*** **♂-contribution**
	*H*	Apo	Apo	Parthenogenetic	1	2	2*n* + 0*n*/2*n* + 4*n*	Apomictic, 4*n* ♂-contribution, 1 polar nucleus fertilised
**2.5**	***I***	**Apo**	**M**	**Parthenogenetic**	**2**	**1**	**2*****n*** **+ 0*****n***/**4*****n*** **+ 1*****n***	**Apomictic, 1*****n*** **♂-contribution**
**2**	*J*	M	M	Parthenogenetic	1	1	1*n* + 0*n*/1*n* + 1*n*	Haploid parthenogenesis, 1*n* ♂-contribution, 1 polar nucleus fertilised
	*K*	Apo	M/Apo	Parthenogenetic	1	2/1^a^	2*n* + 0*n*/2*n* + 2*n*	Apomictic, 2*n* ♂-contribution, 1 polar nucleus fertilised
**1.67**	***L***	**Apo**	**M**	**Sexual**	**2**	**1**	**2*****n*** **+ 1*****n***/**4*****n*** **+ 1*****n***	**♀-Apomeiotic and sexual, 1*****n*** **♂-contribution**
**1.5**	***M***	**M**	**M**	**Sexual**	**2**	**1**	**1*****n*** **+ 1*****n***/**2*****n*** **+ 1*****n***	**♀-Meiotic and sexual, 1*****n*** **♂-contribution**
	*N*	Apo	Apo	Sexual	2	1	2*n* + 2*n*/4*n* + 2*n*	♀-Apomeiotic and sexual, 2*n* ♂-contribution
	*O*	Apo	M	Parthenogenetic	1	1	2*n* + 0*n*/2*n* + 1*n*	Apomictic, 1*n* ♂-contribution, 1 polar nucleus fertilised
1.33	*P*	M	Apo	Sexual	2	1	1*n* + 2*n*/2*n* + 2*n*	♀-Meiotic and sexual, 2*n* ♂-contribution
1	*Q*	M	M	Sexual	1	1	1*n* + 1*n*/1*n* + 1*n*	♀-Meiotic and sexual equal ♀ and ♂-contribution to embryo and endosperm
	*R*	M	Apo	Sexual	1	1	1*n* + 2*n*/1*n* + 2*n*	
	*S*	Apo	M	Sexual	1	1	2*n* + 1*n*/2*n* + 1*n*	♀-Apomeiotic equal ♀ and ♂-contribution to embryo and endosperm
	*T*	Apo	Apo	Sexual	1	1	2*n* + 2*n*/2*n* + 2*n*	

‘Peak index’ refers to the expected endosperm/embryo ploidy ratio. ‘pw’ provides an identification of each pathway. ‘*N* polar nuclei’ and ‘*N* sperm’ list the numbers of these elements contributing to the endosperm. ‘Embryo/endosperm ploidy’ provides for both the embryo and the endosperm the female (first value) and male (second value) genomic contribution. ‘Apo’ and ‘M’ means apomeiotic and meiotic origin, respectively, of female (♀) and male (♂) gametophytes. *n* refers to the holoploid chromosome number notwithstanding the generative ploidy of an individual. Equal ploidy of female and male plants is assumed. Cytological pathways observed within the present study most probable based on the available cyto-embryological literature evidence are in bold type.

a The specified diploid male contribution alternatively may be achieved by contributing two reduced sperm or a single unreduced sperm.

### Progeny survey

To discriminate between the apomictic and sexual origin of seeds with 2*n* embryos and 3*n* endosperm (pathways *M* and *O*) the F_1_ generation was genotyped to test for its recombinant vs nonrecombinant origin. Twenty fruitlets per accession were germinated and grown until two or three leaves had developed. Diploidy (2*n*) of the F_1_ was verified by flow cytometric analysis of petioles as described earlier. Depending on the success of germination we chose 5–10 seedlings per accession for analysis. Total DNA was isolated from silica gel-dried seedlings using the NucleoSpin Plant 96 II extraction kit (Macherey-Nagel, Düren, Germany). An AFLP analysis was conducted using the protocol of Vos *et al*. (1995) with few modifications as described in Paule *et al*. (2011) and employing *Eco*RI-AGG [NED]/*Mse*I-CTC, *Eco*RI-AAC [6-FAM]/*Mse*I-CTT, *Eco*RI-AGC [VIC]/*Mse*I-CTG as three selective primer pairs. Differentially fluorescence labelled PCR products and GS600 LIZ size standards (Applied Biosystems, Foster City, CA, USA) were multiplexed and fragments were separated on a 3730 DNA Analyser (Applied Biosystems). A total of 94 samples and 2 repeat samples were analysed. Raw data were visualised and scored using genemarker version 1.95 (SoftGenetics, State College, PA, USA) and exported as a presence/absence matrix. Recombination and clonality within the F_1_ was estimated using genotype version 1.1 (Meirmans & Tienderen, 2004) based on pairwise differences between pairs of genotypes. Separate threshold values were applied separately for each species analysed, calculated on the basis of the estimated genotyping error and the number of observed AFLP fragments.

## Results

### Performance of the FCSS

**Figure 1:**
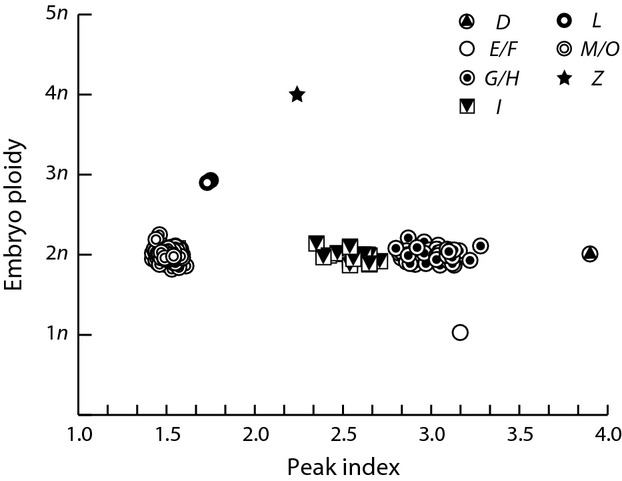
Association of peak indices with embryo ploidy observed in 345 Potentilleae seeds exhibiting embryo and endosperm signals. Symbols refer to seven different cytological pathways of seed formation, as described in Table 1, and an additional apomictic pathway *Z* involving a spontaneous duplication of the embryo sac ploidy.

A clear fluorescence peak of embryo nuclei was obtained for 377 seeds, representing 79.0% of morphologically well-developed seeds subjected to FCSS. At least one additional peak was observed for 346 seeds. Peaks of about double the fluorescence intensity of the embryo peak (1.93–2.11) were observed in 64 seeds and interpreted to represent embryo nuclei in G2 phase (or endopolyploidy). This interpretation is based on the co-occurrence of an additional third peak of a fluorescence intensity expected for the endosperm in all but one of these seeds. Hence, both distinct embryo and endosperm signals were observed in 345 seeds. Inferred peak indices ranged from 1.42 to 3.90, but with discontinuous distribution (Fig. 1). Signals for endosperm nuclei in G2-phase were observed in seven seeds.

The number of embryo nuclei counted per sample and the variation coefficient (CV) of embryo peaks ranged between 320 and 6289 (mean 1676 ± 1023 SD) and 2.28–8.29 (4.70 ± 1.02) for the DAPI-stained samples and between 69–4706 (1119 ± 1092) and 2.83–9.22 (4.47 ± 1.22) for the PI-stained samples. Corresponding values for the endosperm were 22–627 counts (171 ± 121), CV 2.21–6.23 (3.78 ± 0.76) for the DAPI stain and 33–274 counts (85 ± 39), CV 2.37–8.72 (4.78 ± 1.39) for the PI stain.

### Ploidy levels of adults, embryos and seedlings

The karyological survey revealed diploidy (2*n* = 2*x*) and polyploidy (2*n* = 4*x* to 12*x*) for five and eight species, respectively (Table 1). The ploidy level of *Sibbaldia tridentata* could not be established because of failed chromosome counting. Of the embryos 1, 372, 3 and 1 were *n*, 2*n*, 3*n*, and 4*n*, respectively, as inferred from the embryo to mother ratios of relative fluorescence intensities of 1.00, 2.00 ± 0.03 SD, 2.96 ± 0.09 SD and 4.02 (Fig. 1). Hence, 1.34% of embryo formations involved a change in cytotype including a single haploidization and four polyploidization events. Fluorescence ratios of seedlings deviated on average by 4.2% (range 0–16.8%) from that of their mothers, indicating that the germinated F_1_s recovered the 2*n* parental genome.

### Observed cytological pathways of seed formation

**Figure 2:**
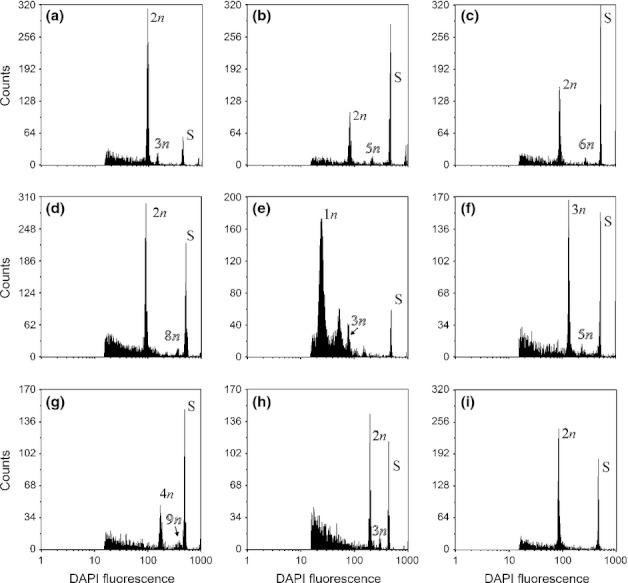
Flow cytometry of Potentilleae seeds representing eight pathways of seed formation defined by a particular combination of embryo ploidy and endosperm/embryo fluorescence ratio (see Table 2). Labels indicate the ploidy of the embryo and endosperm, the standard signal is indicated by S. Unlabeled peaks in (e) are embryo and endosperm nuclei in G2 phase. (a) Regular sexuality (pathway *M*); (b–d) seeds derived via the apomictic pathways *I*, *G*, *D*, respectively. The pathways have a binucleate female contribution to the endosperm in common, but differ in the male contribution to the endosperm; (e) haploid parthenogenesis (pathway *E*); (f) fertilization of an unreduced egg cell (pathway *L*); (g) apomictic pathway *Z* characterized by a 4*n* embryo sac; (h) apomixis involving a uninucleate female contribution to the endosperm (pathway *O*) or pathway *M*. (i) Samples exhibiting a single peak only. (a) *Horkeliella purpurascens*; (b–d, f, g) *Potentilla argentea*; (e) *Potentilla micrantha*; (h) *Potentilla indica*; (i) *Argentina anserina*.

Among the 20 hypothetical pathways of seed formation (described in Table 2) six (pairs of) pathways (*D*, *E*/*F*, *G*/*H*, *I*, *L* and *M*/*O*) could be identified (Table 3; Figs 2) but only *G*/*H*, *I* and *M*/*O* occurred in more than two seeds. Hypothetically, 31 samples with single peaks may represent pathway *Q*. However, they do not contribute to the understanding of endosperm formation because no distinction can be made between whether the endosperm was masked by the embryo (peak index 1) or missed. Hence, they are not considered further. An irregular pathway *Z* characterized by a peak index of 2.24 and a 4*n* embryo resulting from a spontaneous duplication of the embryo sac ploidy was observed for a single seed.

**Table 3:** Cytological pathways of seed formation observed in 14 Potentilleae species

	Expected ploidy																
																
Pathway	Embryo	Endosperm	*Argentina anserina*	*Comarum palustre*	*Dasiphora fruticosa*	*Drymocallis arguta*	*Horkeliella purpurascens*	*Potentilla argentea*	*Potentilla calabra*	*Potentilla incana*	*Potentilla indica*	*Potentilla micrantha*	*Potentilla norvegica*	*Potentilla thuringiaca*	*Sibbaldia procumbens*	*Sibbaldia tridentata*	*N* pathways
*D*	2*n*	8*n*						1									1
								3.90									
*E*/*F*	1*n*	3*n*										1					1
												3.16					
*G*/*H*	2*n*	6*n*						30					24	25			79
								3.04					2.94	3.08			
								± 0.06					± 0.11	± 0.06			
*I*	2*n*	5*n*						8					10	2			20
								2.60					2.52	2.55			
								± 0.07					± 0.11	± 0.02			
*L*	3*n*	5*n*						2									2
								1.74									
								± 0.01									
*M*	2*n*	3*n*	20	19	16	24	28		14	26		23			29	21	220
			1.52	1.49	1.51	1.51	1.51		1.50	1.53		1.54			1.52	1.52	
			± 0.03	± 0.05	± 0.05	± 0.02	± 0.03		± 0.04	± 0.03		± 0.04			± 0.02	± 0.02	
*M*/*O*	2*n*	3*n*									21						21
											1.53						
											± 0.01						
*Z*	4*n*	9*n*						1									1
								2.24									
*Q*?	2*n*	Unknown	1	1		9		5	5				3			7	31
			1	1		1		1	1				1			1	
?	3*n*	Unknown											1				1
													1				
*N* taxon			21	20	16	33	28	47	19	26	21	24	38	27	29	28	377

Pathways are designated according to Table 2 (see also the main text). *Q*? refers to pathway *Q* or alternatively to samples exhibiting a signal for the embryo only. The seed presented in the last line (labelled with ?) showed a 3*n* embryo peak only. ‘*N*’ represents the total sample size for each pathway and taxon. Values are the number of seeds, the mean and standard deviation (SD) of the inferred peak indices.

The AFLP progeny survey was carried out for the 11 species forming seeds via pathway *M*/*O* to identify a sexual origin of seeds. Depending on species, three AFLP primer combinations resulted in 50–114 clearly scorable fragments ranging in size from 56 to 594 bp. The repeatability of the data was 96%. Hence, AFLP phenotypes with a mismatch of two to five AFLP bands were regarded as the same genotype (see Fig. S1). Differences above these thresholds were considered as evidence for a genetically recombined F_1_. Frequency distributions of pairwise genotypic differences among F_1_ individuals suggested genetic recombination for all species tested (i.e. regular sexual pathway *M*) except for *P. indica*. *Potentilla indica* F_1_ individuals differed by a maximum of one fragment making a distinction between pathways *M* and *O* impossible because neither selfing nor homozygosity of the individual can be ruled out (Table 3).

### Taxonomic and phylogenetic distribution of reproductive modes

Apomictic pathways *D, G/H* and *I* were restricted to the so-called ‘core *Potentilla*’ (phylogenetic clade C) and co-occurred with pathway *M*. *Potentilla* species of clade G, as well as all other genera investigated (*Comarum*, *Dasiphora*, *Drymocallis* and *Sibbaldia* – clade A; *Argentina* – clade B; *Horkeliella* – clade E) produced seeds exclusively sexually (i.e. pathway *M*). Apomixis (via pathway *O*) or sexually (pathway *M*) were alternatively inferred for *P*. *indica* (clade F).

In total, 10 out of 14 species showed sexual reproduction via pathway *M* (Table 3). Pathways *G*/*H* and *I* co-occurred in the three remaining species. One of these also showed pathways *D*, *L* and *Z*. Pathways *D, G/H*, and *I* are regular pseudogamous apomictic modes of seed formation and differ in the number of male genomes contributed to the endosperm. Finally, pathway *E/F* (haploid parthenogenesis) was observed in only one seed of *P. micrantha* which otherwise formed seeds via pathway *M*.

### Gamete ploidy variation

Variation in the ploidy of female and male gametes is shown in Table 4. Seeds of *P. indica* were excluded from the statistics as its mode of reproduction remained uncertain. Female and male genomic contributions calculated using the formulae provided in Notes S1 revealed three (*c*. *n*, 2*n*, 4*n*) and four classes (*c*. *n*, 2*n*, 3*n*, 4*n*), respectively. As predicted by inferred cytological pathways, sexual species mainly produced *n* gametes, while apomicts produced 2*n* egg cells and received a male contribution of *n* (21%), 2*n* (78%) or rarely 4*n* (1%) to the endosperm. Male and female gametes exhibited similar deviations from *n* in sexual species (CV = 0.083 and CV = 0.068 for sperm and egg cells, respectively). Ploidy variation in parthenogenetically derived seeds, by contrast, was considerably higher for sperm (CV = 0.154 and CV = 0.093 for *n* and 2*n* genomic contributions, respectively) compared to egg cells (CV = 0.032). Ploidy variation in sperm of odd-ploid species (*Potentilla thuringiaca*, 2*n* = 9*x*: CV = 0.066) was in the range of that in even-ploids (*Potentilla argentea*, 6*x*: CV = 0.058; *Potentilla norvegica*: 10*x*, CV = 0.095).

**Table 4:** Ploidy variation in female and male gametes inferred for seeds with parthenogenetically and sexually derived embryos from 13 taxa of the tribe Potentilleae

Sexual origin	*N* seeds	Mean *n* ± CV	Range *n*
Female gametes			
*n*	220	1.03 ± 0.07	0.81–1.19
2*n*	2	2.15 ± 0.02	2.11–2.18
Male gametes			
*n*	222	0.96 ± 0.08	0.73–1.22
2*n*	0	–	–
Parthenogenetic origin			
Female gametes			
*n*	1	1.03	–
2*n*	100	2.00 ± 0.03	1.87–2.21
Male gametes			
*n*	21	1.10 ± 0.15	0.75–1.36
‘2*n*’	78	2.04 ± 0.09	1.62–2.38

Seeds of *Potentilla indica* were excluded as its mode of reproduction remained uncertain. Ploidies are calculated using the formulas detailed in the Supporting Information, Notes S1. Gametes are categorized according to ploidy (*n* and 2*n*). In seeds with parthenogenetically derived embryos single unreduced male gametes (2*n*) cannot be distinguished from the involvement of two reduced (*n* + *n*) sperm nuclei and have to be understood as a genomic contribution. 3*n* and 4*n* contributions were inferred only for single seeds and are, hence, not considered. CV, coefficient of variation.

## Discussion

### Applicability of FCSS in the Potentilleae

The low amount of endosperm tissue – although higher than suggested by Martin (1946), Corner (1976) and Kalkman (2004) – and considerable background noise demanded a modification of the laboratory protocol of Matzk *et al*. (2000). Modifications involved: rejection of signals other than nuclei using the side scatter (PI-stained seeds); reduction of the DAPI-concentration in the staining buffer by a factor of 100; removal of the testa and fruit wall reducing the fluorescence signal of nonspecifically stained particles; improvement of the efficiency of nuclei extraction by ultrasonication of the chopped sample; elongation of the extraction time; and logarithmic amplification of the fluorescence signal, resulting in higher endosperm peaks. Using this modified protocol, distinct endosperm and embryo fluorescence peaks were obtained for 91.5% of seeds, representing all species studied covering all major evolutionary lineages in the Potentilleae (Eriksson *et al*., 1998, 2003; Dobeš & Paule, 2010). Thus, our data suggest a tribe-wide applicability of the FCSS procedure, but with methodological limits (see also next section), which are particularly critical for the detection of apomixis. Endosperms of apomicts had a lower average number of nuclei (mean 85.5 ± 48.4 SD nuclei calculated for DAPI-stained samples; count ratio 6.61% ± 3.10) compared with sexual species (176.9 ± 123.7; count ratio 13.10% ± 4.59) presumably owing to higher ploidy. Ploidy thus has typically a positive and a negative correlation with cell size (Müntzing, 1936; Stebbins, 1971; Gregory, 2001) and growth rates of tissues (Cavalier-Smith, 1978; Levin, 1983), respectively, potentially decreasing the number of nuclei in the high-polyploid endosperms of the apomicts.

### Mathematical inference of male and female genomic contributions and associated limitations

We calculated the male and female genomic contribution for seeds receiving a binucleate female contribution to the endosperm. The calculation is based on the finding that under this condition seeds with peak indices ranging from 1 to 2 and from 2 to ∞ have sexually and parthenogenetically derived embryos, respectively, independent of the origin (meiotic vs apomeiotic) of the male and female gametophytes and the numbers of contributing sperm (see Notes S1 for reasoning and the mathematical framework). The calculation of the male and female genomic contribution allows for a direct assessment of the origin of embryos and the endosperm (without consulting Table 2), that is, basically whether embryo sacs are reduced or unreduced (or even of increased ploidy) and whether embryos are of zygotic or parthenogenetic origin. Furthermore, our approach is applicable in cases of gamete ploidy variation caused by meiotic disturbances (Müntzing, 1931; Rutishauser, 1948; Asker, 1970a) and intercytotype crosses (Müntzing & Müntzing, 1945; Smith, 1963a; Nosrati *et al*., 2010) resulting in a multitude of unpredictable embryo ploidy–peak index combinations.

The vast majority of gametes were *n* and 2*n,* suggesting an origin via both meiosis or apomeiosis from parents of equal ploidy. As an exception, a single 4*n* female contribution to the embryo was detected for *P. argentea*, which can be explained by spontaneous genome duplication (in an apomictically formed seed), as already observed in *Potentilla* (Müntzing & Müntzing, 1943). Furthermore, our data superficially suggest cytological irregularities in the formation of pollen in apomicts, as commonly reported for *Potentilla* species (Müntzing, 1931; Gentscheff & Gustafsson, 1940). Ploidy variation for *n* (CV = 0.154) and 2*n* (CV = 0.093) male contributions in apomicts thus was about five and three times higher, respectively, than for apomeiotically formed egg cells (0.032). However, these differences can be explained by a systematic error in the estimation of peak indices, which does not influence ploidy inference of egg cells, but multiplies the corresponding error in inference of 2*n* and *n* male contributions three- and five-times, respectively (Fig. S2). Thus, the estimation of sperm cell ploidy strongly depends on a precise flow cytometric estimation of endosperm and embryo signals, particularly in apomicts. Factors potentially influencing the accuracy of peak index estimation involve the performance of the flow cytometer, tissue-specific differential expression of secondary metabolites (e.g. polyphenolics; Bate-Smith, 1961; Tomczyk *et al*., 2010) or DNA degeneration owing to shrivelled tissues in embryo and endosperm.

The systematic error of the peak index also magnifies the error in the estimation of ploidy of *n* male and *n* female gametes contributing to seeds with sexually derived embryos (2*n* embryo and 3*n* endosperm; Fig. S2). However, the application of our approach to sexually derived seeds collected from different cytotypes of *P. puberula* revealed an additional increase in chromosome number variation among female gametes of high polyploids compared with tetraploids, suggesting disturbance of meiosis (Ch. Dobeš, A. Milosevic, S. Scheffknecht, unpublished). This finding highlights the potential of the new method for a better understanding of the cytology of seed formation. The approach is further considered useful for the determination of male gametophyte ploidy in place of flow-cytometric measurement of pollen, which is methodologically demanding (e.g. Pan *et al*., 2004; Stehlik *et al*., 2007; Takamura & Yoshimura, 2007), although pollen ploidies compatible in a cross only can be inferred from seeds.

### Cytology of the male genomic contribution to the endosperm remained hypothetical in apomicts

The endosperm in apomicts received an *n* or 2*n* male contribution in 21% and 78% of seeds, respectively. However, the cyto-embryological literature refers to the involvement of a single reduced sperm (Gentscheff & Gustafsson, 1940; Håkansson, 1946; Smith, 1963b). Variation in the number of sperm contributing to the endosperm in pseudogamous apomicts was reported from the rosaceous genera *Crataegus* (Talent & Dickinson, 2007) and *Sorbus* (Jankun & Kovanda, 1988). In *Crataegus* 60% and 25% of the apomictically formed seeds received one and two sperm nuclei, respectively. Such variation indicates that the ratio of maternal to paternal genomes in the endosperm is not constrained to 2 *m* : 1*p* in these genera as it is in most angiosperms (Vinkenoog *et al*., 2003). This was hypothetically explained by diversion of the second sperm to the central cell by a hormonal attractant expressed by the central cell but suppressed in the egg cell (Talent & Dickinson, 2007). This explanation might particularly apply to *Potentilla* given that the majority of central cells received a 2*n* male contribution (Table 3). An alternative explanation may be irregularities in the male meiosis resulting in the formation of both reduced and unreduced sperms. Unexpectedly, ploidy variation in sperm was not higher in odd-ploid (*P. thuringiaca*) than in even-ploid apomicts (*P. argentea* and *P. norvegica*). Interestingly, 92.6% of seeds received a 2*n* male contribution to the endosperm in odd-ploid *P. thuringiaca* compared with 63.2–63.8% in the even-ploids. These high proportions may indicate an apomeiotic formation of pollen, but this assumption has to be tested on a larger sample of odd-ploid and even-ploid cytotypes, which are both frequently observed in apomictic *Potentilla* species (e.g. Dobeš, 1999).

### Number of polar nuclei involved in endosperm formation and applicability of FCSS in Potentilleae

Pathways *G*/*H* represented the most frequent apomictic pathway of seed formation observed in our data. Both pathways differ in the involvement of one and two polar nuclei in endosperm formation, respectively and both give rise to seeds with 6*n* endosperms formed either by 4*n* female and 2*n* male contributions or vice versa (Table 2). However, pathway *H* is unlikely as it requires three processes with little literature-based cytological and embryological evidence for the Potentilleae: male apomeiosis and involvement of a single polar nucleus, and fertilized by two sperm. By contrast, a single element, unexpected, from cyto-embryology has to be assumed in the case of pathway *G*: male apomeiosis or, alternatively, double-fertilization of the central cell. Furthermore, pathway *G* differed from pathway *I* (as found in the same species) in a single cytological element only (Table 5). Additional pathways observed in apomicts were *D*, *L* and *Z,* which can be derived from pathway *I* by the change of one or two (in the case of pathway *D*) cytological elements and share a binucleate female contribution to the endosperm. Apomictic pathways co-occurring within a species thus are cytologically more closely related to each other and more likely according to the cyto-embryological literature record under the assumption of a binucleate female contribution to the endosperm compared with involvement of a single polar nucleus (Table 5).

**Table 5:** Comparison of the cytology of pathways co-occurring in apomictic *Potentilla* species

Peak index	Pathway	Gametophyte origin		Embryo development	Endosperm origin	
	
		♀	♂		*N* polar nuclei	*N* sperm
4	*D*	Apo	Apo	Parthenogenetic	2	2
3	*G*	Apo	M	Parthenogenetic	2	2
		Apo	Apo	Parthenogenetic	2	1
	*H*	Apo	Apo	Parthenogenetic	1	2
2.5	*I*	Apo	M	Parthenogenetic	2	1
1.67	*L*	Apo	M	Sexual	2	1
2.25	*Z*	Apo	M	Parthenogenetic	2	1

Major pathway *I* is characterised by an explicit combination of cytological elements all expected from the cyto-embryological literature record. Pathways *G* and pathway *H* could not be distinguished from each other by FCSS but differ from co-occurring pathway *I* by one or three elements, respectively, making pathway *H* unlikely. The remaining pathways *D*, *L* and *Z* can be derived from pathway *I* by changing one or two elements. Pathways most closely related to pathway *I* all share a binucleate female contribution to the endosperm. ‘Apo’ and ‘M’ means apomeiotic and meiotic origin of female (♀) and male (♂) gametophytes. Cytological elements expected based on the cyto-embryological literature record are underlined.

In case of pathways *M*/*O*, the genetically recombined F_1_ for 10 out of the 11 species tested has revealed that seeds arise via regular sexuality (i.e. pathway *M*; thus dismissing pathway *O*). In total, pathway *M* occurred in 63.8% of the seeds analysed that exhibited endosperm signals and is characterized by the contribution of two polar nuclei and a single reduced sperm to the endosperm, reflecting the common cytology of sexual seed formation in angiosperms (Rutishauser, 1967; Asker & Jerling, 1992) and exemplarily documented in the genus *Potentilla* (Rutishauser, 1945; Håkansson, 1946; Czapik, 1961, 1962).

The most parsimonious interpretation of the combined evidence on both sexual and pseudogamous apomictic seed formations in the Potentilleae favours involvement of two polar nuclei in the origin of the endosperm in the majority of the taxa studied. Such a binucleate female contribution to the endosperm was inferred for all segregate genera studied (*Argentina*, *Comarum*, *Dasiphora*, *Drymocallis* and *Sibbaldia*) and three major evolutionary lineages of the genus *Potentilla sensu stricto* (Dobeš & Paule, 2010): ancient clade G, the derived core group (clade C) and its next sister, clade E (represented by *Horkeliella purpurascens*). The finding is crucial as a prerequisite to discriminate between these two principal modes of seed formation using FCSS (Matzk *et al*., 2000). However, the genetically nonrecombinant F_1_ individuals of *P. indica* (clade F corresponding to the series Tormentillae; Wolf, 1908) indicated that seeds may have originated via pathway *O* involving only a single polar nucleus in endosperm formation. Nevertheless, a nonrecombinant F_1_ is also expected for homozygous sexual parents. High degrees of homozygosity arise following repeated cycles of selfing (i.e. strict autogamy) and in genetically highly depauperate populations (Hartl & Clark, 1997). Both explanations are, however, not in line with the floral syndrome of the species typical for outcrossers (Naruhashi & Sugimoto, 1996) and intraspecific genotypic variation (Ontivero *et al*., 2000). Controlled crosses (Debes *et al*., 2011) and embryological studies (carried out on several other species of the Tormentillae; Schwendener, 1969; Czapik, 1975) also were inconclusive and the authors speculated about the coexistence of apomixis and sexuality within individuals. Hence, the Tormentillae are a good example to demonstrate the difficulties in inferring reproductive modes. Unintended selfing that may occur in controlled crosses (Schwendener, 1969), as well as maternal inheritance of markers in progeny tests (Debes *et al*., 2011), may therefore be erroneously interpreted as apomixis. The determination of actual reproductive modes may be further hampered by the co-occurrence of meiotic and apomeiotic embryo sac initials in single flowers as well as by limitations in the currently available methodology for documenting all cytological elements in seed formation using a traditional embryological approach (Schwendener, 1969; Czapik, 1975).

Our results may not allow a generalization because only 14 out of a few hundred species in the tribe Potentilleae were investigated. Thus, our FCSS-based inferences are conclusive only if the cytology of seed formation is widely conserved within the tribe. Unfortunately, variation in the number of polar nuclei contributing to the endosperm among phylogenetic lineages and even among individuals within one species cannot be ruled out completely. However, the cytological pathways of 22 additional Potentilleae species in an unpublished data set show a distribution across the phylogeny that is fully congruent with that presented in this study (e.g. pathway *M*/*O* was observed in all lineages, apomixis involving two polar nuclei was proven for clade C only). The phylogenetic distribution of seeds with diploid embryo and triploid endosperm strengthens the evidence that apomictic pathways differing in the number of polar nuclei contributing to the endosperm have not evolved in parallel within a single lineage. Specifically, pathway *M*/*O* was also observed for two species from the Tormentillae (*P. erecta* and *P. reptans*), for which embryology suggested a high probability of apomictic reproduction (Forenbacher, 1914; Schwendener, 1969). Our results suggest that the involvement of two polar nuclei in endosperm formation is phylogenetically conserved in the tribe Potentilleae, although a uninucleate contribution – preventing the application of our approach – could not be excluded for the series Tormentillae.

## Supporting Information

**Fig. S1** Frequency distribution of pairwise genotypic differences between individuals.

**Fig. S2** Influence of the systematic error of the peak index on inference of female and male genomic contributions.

**Notes S1** Mathematical derivation of male and female genomic contributions to embryo and endosperm based on the embryo ploidy and the peak index
