# Supplementary material for: Prospects and limits of the flow cytometric seed screen – insights from Potentilla sensu lato (Potentilleae, Rosaceae)
Source: New Phytol. 2013 Feb 21;198(2):605–16. doi: 10.1111/nph.12149 (PMC3618378; doi:10.1111/nph.12149)

Supporting Information Fig. S1

**Fig. S1** Frequency distribution of pairwise genotypic differences (*x*-axis) between pairs of individuals (*y*-axis). Red square indicates the threshold value based on the 96% repeatability counted for each sample separately.

Ptl2650 *Drymocallis arguta* - 50 AFLP fragments

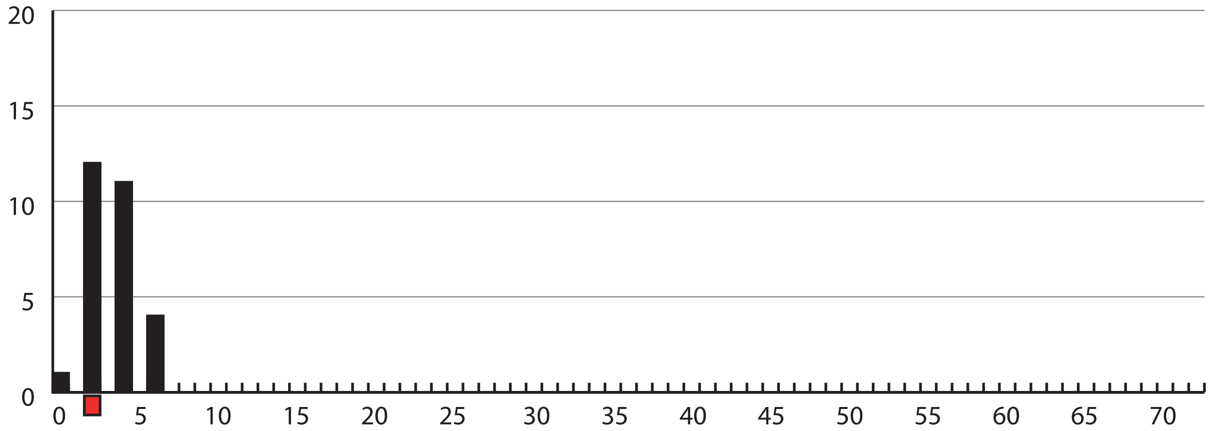


Ptl4731 *Potentilla calabra* - 73 AFLP fragments

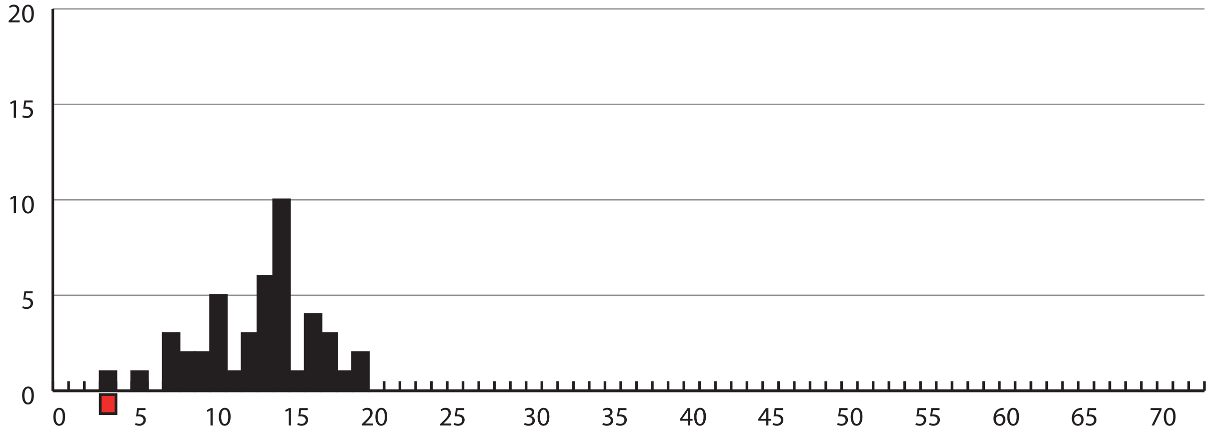


Ptl8202 *Potentilla indica* - 78 AFLP fragments

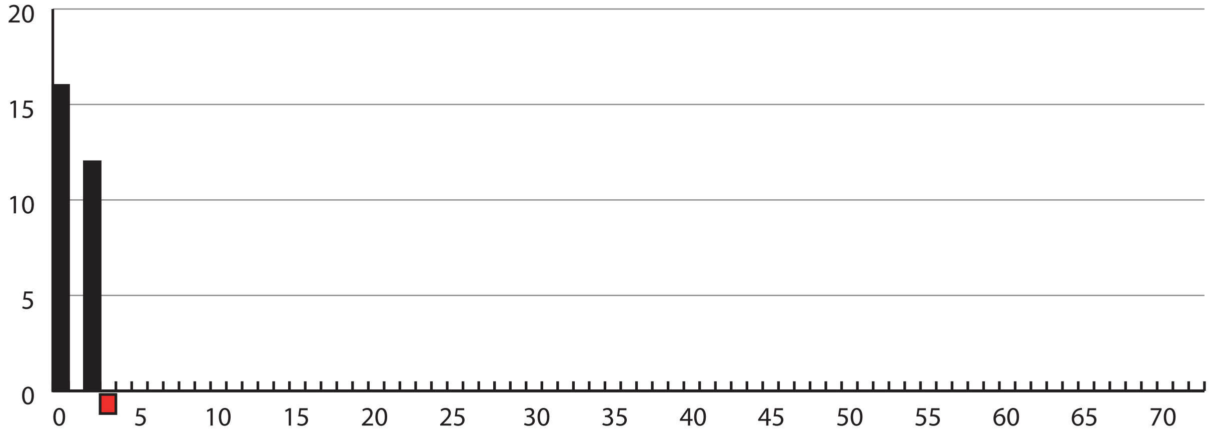

Ptl8224 *Potentilla micrantha*- 70 AFLP fragments

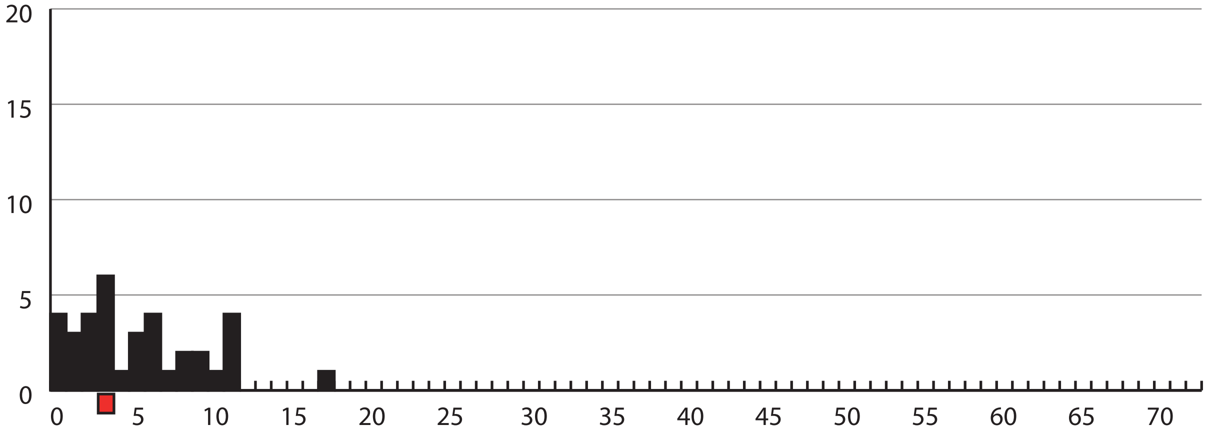


Ptl8249 *Potentilla incana* - 105 AFLP fragments

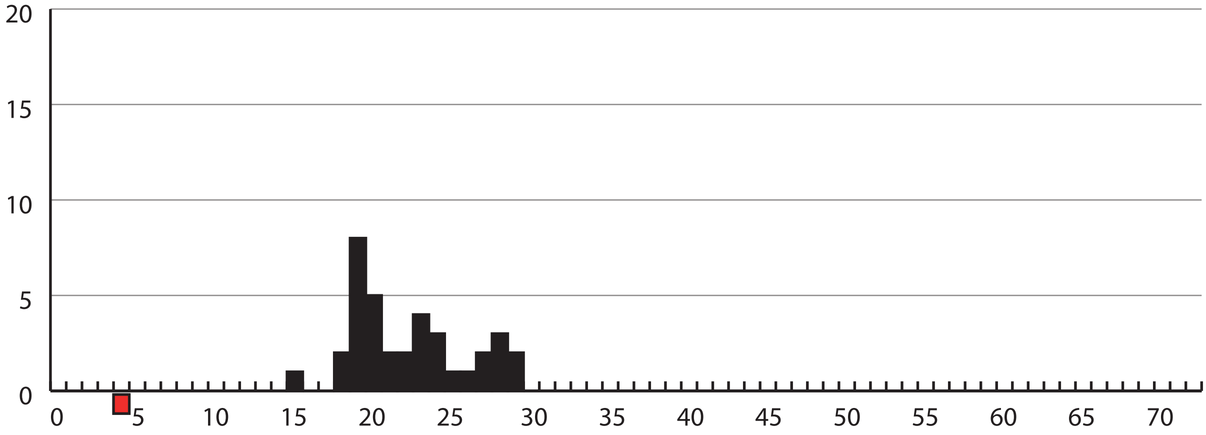


Ptl8407 *Sibbaldia procumbens* - 71 AFLP fragments

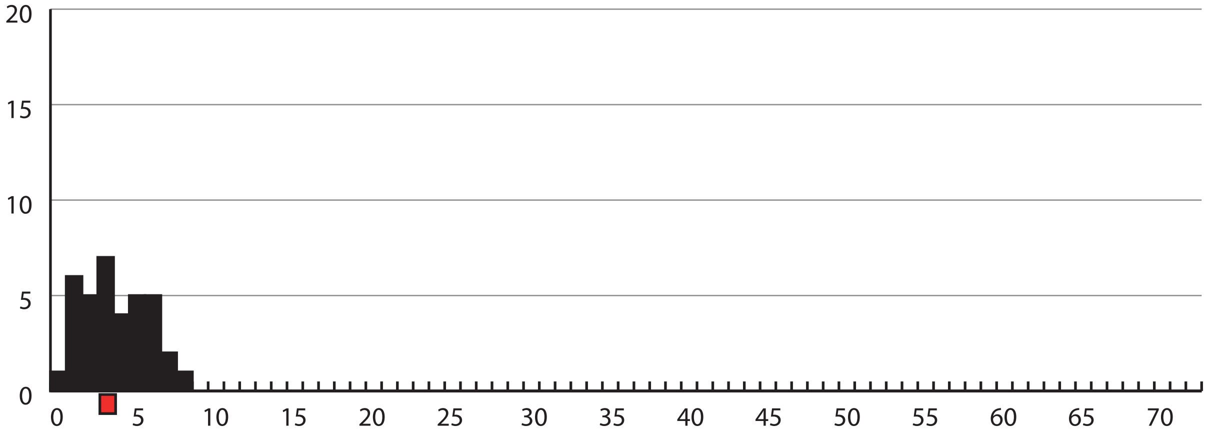

Ptl8410 *Comarum palustre* - 108 AFLP fragments

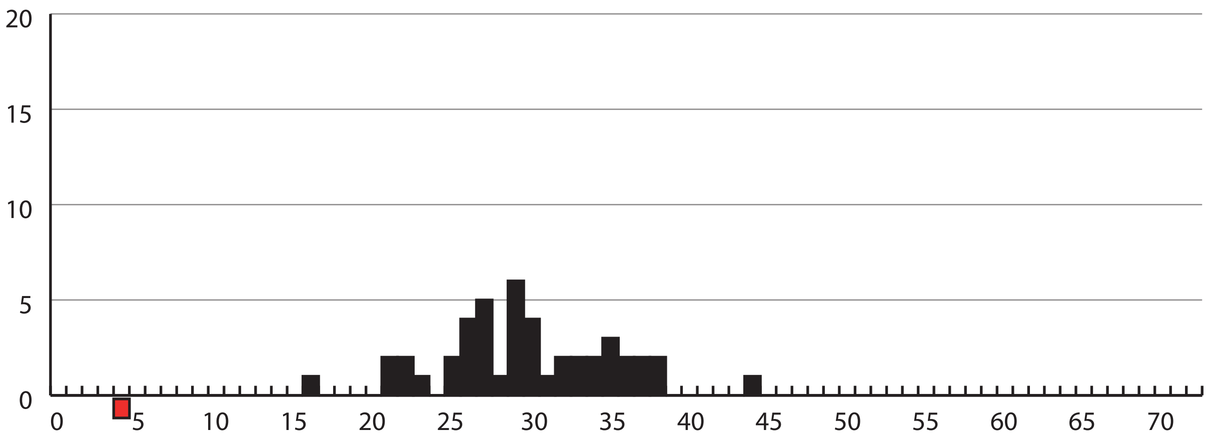


Ptl8411 *Dasiphora fruticosa* - 69 AFLP fragments

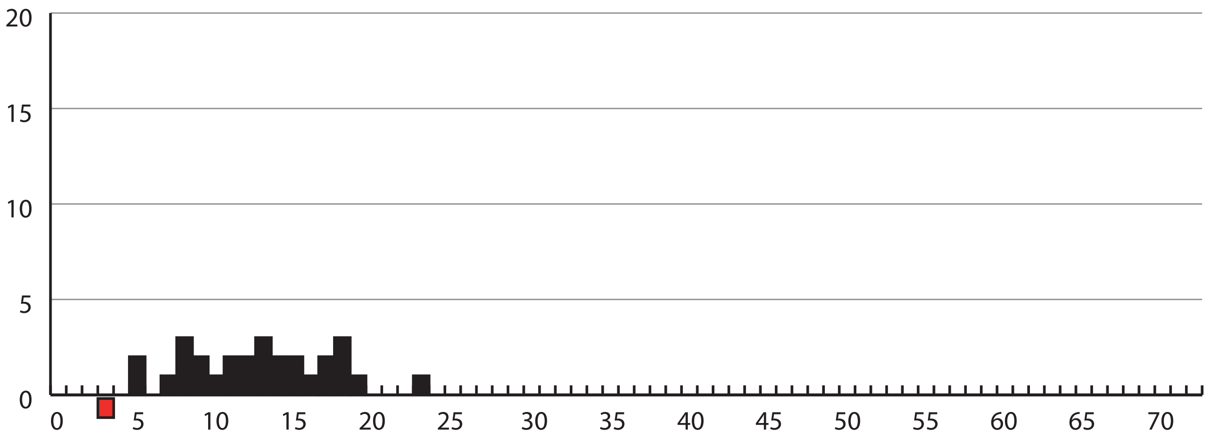


Ptl8413 *Sibbaldia tridentata* - 114 AFLP fragments

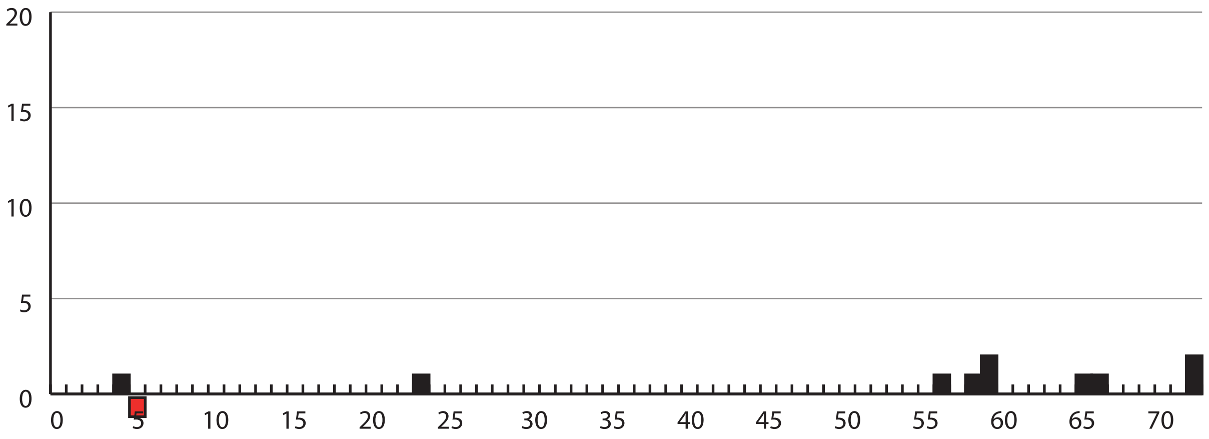

Ptl8415 *Argentina anserina* - 72 AFLP fragments

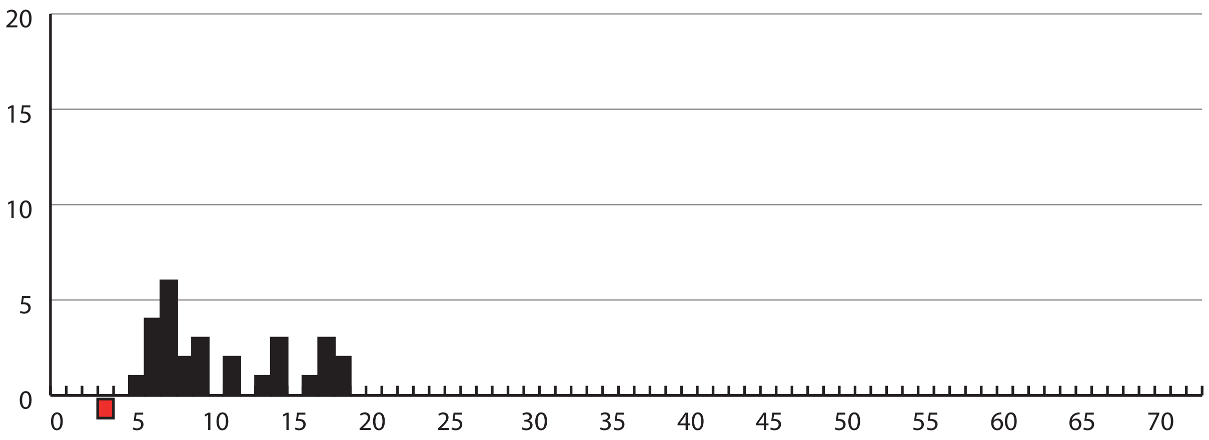


Ptl8418 *Horkeliella purpurascens* - 73 AFLP fragments

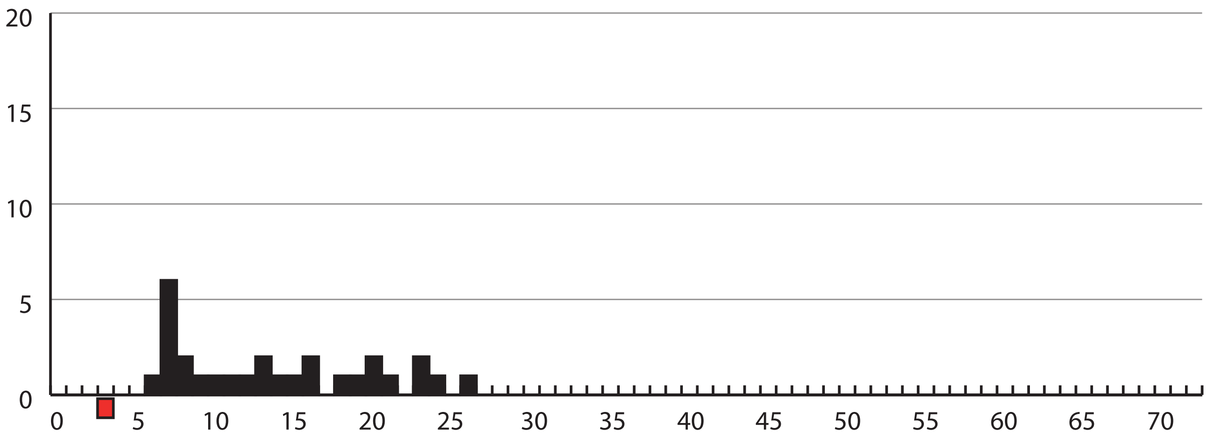

Supplement: Supplementary file 1 [file nph0198-0605-SD1.pdf]
