# Supplementary material for: Prospects and limits of the flow cytometric seed screen – insights from Potentilla sensu lato (Potentilleae, Rosaceae)
Source: New Phytol. 2013 Feb 21;198(2):605–16. doi: 10.1111/nph.12149 (PMC3618378; doi:10.1111/nph.12149)

Supporting Information

**Fig. S2** Influence of the error in estimating the peak index on the inference of female and male genomic contributions in apomictically- and sexually-derived seeds. The female genomic contribution equals the ploidy of the embryo sac (and the egg cell). The male genomic contribution is the number of genomes contributed to the endosperm by one or two sperm in apomictically-derived seeds and the ploidy of the sperm in sexually-derived seeds.

Apomictically-derived seeds: The relationship applies to endosperms receiving a bi-nucleate (4*n*) female and an *n* or 2*n* male genomic contributions (corresponding to peak indices of 2.5 and 3, respectively). The ploidy of the embryo sac equals the ploidy of embryos – which themselves are directly inferred from the embryo/standard fluorescence ratio – and is, hence, not related to the peak index (symbolized by diamonds). In contrast, the systematic error of the peak index implies a 3- and 5-fold error in ploidy inference of 2*n* (squares) and *n* (circles) male contributions, respectively: e.g. a 20% deviation of the peak index results in a 60% and 100% deviation of 2*n* and *n* male contributions.

Sexually-derived seeds: The relationship applies to endosperms receiving a bi-nucleate (2*n*) female contribution and an *n* male genomic contribution (corresponding to a peak index of 1.5). The systematic error of the peak index means a 3-fold error in ploidy inference of *n* female and male contributions (squares).


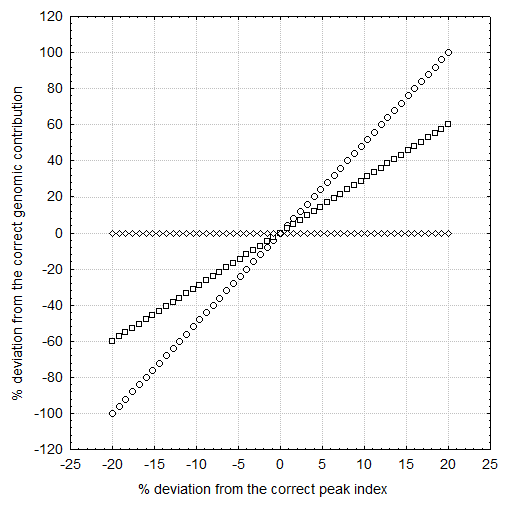

Supplement: Supplementary file 2 [file nph0198-0605-SD2.docx]
