# Supplementary material for: Prospects and limits of the flow cytometric seed screen – insights from Potentilla sensu lato (Potentilleae, Rosaceae)
Source: New Phytol. 2013 Feb 21;198(2):605–16. doi: 10.1111/nph.12149 (PMC3618378; doi:10.1111/nph.12149)
Supplement: Supplementary file 3 [file nph0198-0605-SD3.docx]

**Supporting Information Notes S1**

**Notes S1** Mathematical derivation of the male and female genomic contributions to embryo and endosperm based on the embryo ploidy and the peak index.

**A) Differentiation between the sexual and parthenogenetic origin of the embryo**

The ratio of endosperm to embryo ploidy (i.e. peak index) can be used to distinguish between different reproductive modes of embryo formation. Peak indices lower and higher than 2 indicate the sexual and parthenogenetic origin of the embryo, respectively. Theoretical ranges of peak indices are described by the following formulas, which are based on the assumptions of a bi-nucleate female contribution to the endosperm and fertilisation of the polar nuclei.

| **Origin embryo** | **Parthenogenesis** | **Sexuality** |
| --- | --- | --- |
| embryo ploidy | ♀ | ♀ + ♂ |
| endosperm ploidy | 2 × ♀ + ♂ | 2 × ♀ + ♂ |
| depending on the ploidy of parental individuals the peak index may range from | | |
| lowest peak index | (2 × ∞♀ + 1♂) / ∞♀ ≥ 2 | (2 × 1♀ + ∞♂) / (1♀ + ∞♂) ≥ 1 |
| highest peak index | (2 × 1♀ + ∞♂) / 1♀ ≤ ∞ | (2 × ∞♀ + 1♂) / (∞♀ + 1♂) ≤ 2 |

with

♀ = ploidy of the embryo sac

♂ = male genomic contribution^A^

**B) Calculation of the male genomic contribution^A^ (♂) and embryo sac ploidy (♀)**

Two sets of formulas apply to seeds with sexually- and parthenogenetically-derived embryos. The number of contributing holoploid female genomes is indicated by *n*^B^ (the haplophasic chromosome number). The female genomic contribution to the embryo equals the embryo sac ploidy. The female genomic contribution to the endosperm is twice the embryo sac ploidy. Calculations base on the assumption of a bi-nucleate female contribution to the endosperm and fertilisation of the polar nuclei (see A).

| **Origin of the embryo** | **Origin embryo sac (=female gametophyte)** | **Ploidy embryo** | **Ploidy endosperm**^C^ | **Calculation of ♀ and ♂** |  |
| --- | --- | --- | --- | --- | --- |
| **Sexual** | meiotic | n+♂ | 2n+♂ |  | |
| Peak index = (1, ≤ 2) | (=regular sexuality) |  |  |  | |
|  | apomeiotic | 2n+♂ | 4n+♂ |  | |
|  |  |  |  |  | |
|  | generalised form | ♀+♂ | 2♀+♂ | **♀ = ploidy endosperm - ploidy embryo*** | |
|  |  |  |  | **♂ = ploidy embryo - ♀ = ploidy endosperm - 2♀** |  |
|  |  |  |  |  | |
| **Parthenogenetic** | meiotic | n | 2n+♂ |  | |
| Peak index ≥ 2 | = (haploid parthenogenesis) | |  |  | |
|  | apomeiotic | 2n | 4n+♂ |  | |
|  | (= apomixis) | | |  | |
|  | generalised form | ♀ | 2♀+♂ | **♀ = ploidy embryo** | |
|  |  |  |  | **♂ = ploidy endosperm - 2 × ploidy embryo**** |  |

**Derivation of the formulas**

* Sexual embryo origin

ploidy embryo = ♀ + ploidy endosperm - 2♀ = - ♀ + ploidy endosperm

♀ = ploidy endosperm - ploidy embryo

** Parthenogenetic embryo origin

ploidy embryo = (ploidy endosperm - ♂)/2

2 × ploidy embryo = ploidy endosperm - ♂

♂ = ploidy endosperm - 2 × ploidy embryo

**Notes**:

^A^ The male genomic contribution is the number of male genomes received by each the embryo and endosperm in seeds with sexually-derived embryos or the endosperm only in seeds with parthenogenetically-derived embryos.

^B^ *n* may be replaced by *x* (the chromosome number of the monoploid genome) for mothers of known generative ploidy.

^C^ The ploidy of the endosperm (expressed on the basis of *x* or *n*) has to be calculated from the embryo ploidy and the peak index (endosperm ploidy = embryo ploidy × peak index).
